# Attentional Modulation of Change Detection ERP Components by Peripheral Retro-Cueing

**DOI:** 10.3389/fnhum.2017.00076

**Published:** 2017-02-21

**Authors:** Paula Pazo-Álvarez, Adriana Roca-Fernández, Francisco-Javier Gutiérrez-Domínguez, Elena Amenedo

**Affiliations:** ^1^Department of Clinical Psychology and Psychobiology, Faculty of PsychologySantiago de Compostela, Spain; ^2^Nuffield Department of Clinical Neurosciences, John Radcliffe Hospital, University of OxfordOxford, UK

**Keywords:** change detection, change blindness, ERPs, late positivity, peripheral cues, retro-cueing, visual awareness negativity

## Abstract

Change detection is essential for visual perception and performance in our environment. However, observers often miss changes that should be easily noticed. A failure in any of the processes involved in conscious detection (encoding the pre-change display, maintenance of that information within working memory, and comparison of the pre and post change displays) can lead to change blindness. Given that unnoticed visual changes in a scene can be easily detected once attention is drawn to them, it has been suggested that attention plays an important role on visual awareness. In the present study, we used behavioral and electrophysiological (ERPs) measures to study whether the manipulation of retrospective spatial attention affects performance and modulates brain activity related to the awareness of a change. To that end, exogenous peripheral cues were presented during the delay period (retro-cues) between the first and the second array using a one-shot change detection task. Awareness of a change was associated with a posterior negative amplitude shift around 228–292 ms (“Visual Awareness Negativity”), which was independent of retrospective spatial attention, as it was elicited to both validly and invalidly cued change trials. Change detection was also associated with a larger positive deflection around 420–580 ms (“Late Positivity”), but only when the peripheral retro-cues correctly predicted the change. Present results confirm that the early and late ERP components related to change detection can be functionally dissociated through manipulations of exogenous retro-cueing using a change blindness paradigm.

## Introduction

Under normal circumstances, changes in visual scenes usually produce motion transients, which automatically capture attention in a bottom–up fashion and facilitate the conscious detection of the change (CD). This conscious detection involves a number of steps including, within others, the encoding of the pre-change display, the maintenance of this information for several milliseconds within working memory, and the comparison of the pre and post change displays. However, a failure in any of these processes can lead to what is called change blindness (CB, Rensink et al., 1997; Rensink, 2002). For instance, it is well-documented that eliminating or masking the transients that facilitate CD by their simultaneous occurrence with visual disruptions produces CB, and reveals that the conscious representation of our visual surroundings presents important limitations. Given that unnoticed visual changes in a scene can be readily detected once attention is drawn to them, it has been suggested that attention is crucial to change awareness (Landman et al., 2003). This hypothesis is supported by findings showing that changes are more likely to be detected when they take place in objects that, because of their salience, or because they have been cued before the change, receive preferential attention in a scene (Rensink, 2002).

Change detection studies have, among others, shown experimentally that our conscious experience is limited (Lamme, 2003). However, the nature of conscious perception is difficult to clarify, and has been the center of debate in recent years. In this context, Block (1995, 2005) introduced in the literature two different terms, phenomenal consciousness and access consciousness. The phenomenal consciousness refers to “subjective” sensory experiences, which may be associated with activity in the extrastriate visual areas especially along the ventral visual stream (Lamme, 2003), whereas access consciousness would involve modality-independent areas related to the control of processes such as attention or working memory. However, other theories do not agree with this differentiation between phenomenal and access consciousness (see Dehaene et al., 2006).

Electrophysiological studies on CD (see (Koivisto and Revonsuo, 2010) for a review) have suggested two successive Event-Related Potential (ERP) components, which can be observed when CD and CB trials are compared, as the most reliable and consistent correlates of conscious CD. One earlier component, the visual awareness negativity or VAN, which is a negative amplitude difference that appears at occipito-temporal scalp sites in the N1–N2 latency range after the onset of the detected change, and a later component, called late positivity or LP, which consists in a positive deflection with a broad scalp distribution that appears after 300 ms or later once the change has been detected. Given the different latencies and scalp distributions of VAN and LP components, and following Block's proposal (1995, 2005), it has been suggested that VAN could be the correlate of neural processes occurring when the stimulus/change enters phenomenal visual consciousness whereas the later LP component could be a sign of the stimulus entrance in access consciousness (see Koivisto and Revonsuo, 2010 for a review). Nevertheless, the specific role of both components in visual awareness is still under debate.

Another topic of debate is the relationship between attention and awareness. In the case of change blindness, there is a general consensus on the fact that focal spatial attention is needed in order to perceive the change. In this context it has been generally found that the most well-differentiated forms of spatial attention, endogenous (top-down) and exogenous (bottom-up) attention, are very important for awareness but can have different effects on it (see Chica and Bartolomeo, 2012 for a comprehensive review).

In laboratory conditions, the orienting of endogenous and exogenous attention has been extensively studied using the “Posnerian” cueing paradigm, and its variants. This paradigm uses stimuli that predict the location of detected stimuli (the target), and are called spatial cues. These cues can be central (classically consisting of stimuli presented at fixation, such as arrows) or peripheral (stimuli presented at target locations). Both types of cues can be either informative or uninformative depending on whether they validly predict the location of the subsequently presented target stimuli. Researchers have normally used informative central or peripheral cues to elicit endogenous attentional orienting, while non-predictive peripheral cues have been employed to investigate exogenous spatial attention orienting (see Chica et al., 2007).

In the context of change detection, behavioral studies have investigated the role of attention on awareness, and it has been probed that cueing the relevant item before the potential change protects from CB (see Lamme, 2003). It has been suggested that in change detection experiments subjects may try to “sense” the change across the display as a whole, circumstance that could be ideal for exogenous attentional capture, which should be detectable as attenuated change blindness to exogenously attended items (Scholl, 2000). Accordingly, it has been observed using a flicker paradigm that late-onset items presented before the pre-change image captured attention exogenously and produced reduction of response times to detected changes in the image that contains it (Scholl, 2000). In line with these results, but using a one-shot paradigm, Smith and Schenk (2008) reported that peripheral cues transiently enhanced awareness at very short cue–change latencies (150 ms). However, these authors found that at 480 ms this effect was abolished. These findings suggest that objects that exogenously attract the observer's attention have a more detailed representation in working memory (Simons and Rensink, 2005) and subsequently decrease change blindness (Scholl, 2000). In a later study, Smith and Schenk (2010) observed that even the use of subliminal peripheral pre-change cues had a significant effect on the detection of changes.

Considering that attention has proved to be an important factor to improve behaviorally the detectability of a change in CB experimental contexts, it results interesting to explore whether this improvement is also evident in the ERP correlates of change detection, VAN and LP. To our knowledge, there is only one study that has explored the electrophysiological effects of attention using a change detection paradigm. Specifically, Koivisto and Revonsuo (2005) investigated whether endogenous spatial attention deployed to the pre-change display affected the electrophysiological correlates of awareness VAN and LP. They presented, simultaneously with the pre-change display, trials with informative central cues (70% validity), trials with invalid cues, and trials with no cue. Behavioral results showed more accurate responses after valid cues and therefore a facilitation of CD. The ERPs to the change display showed an increased negativity around 200 ms (VAN) and an increased positivity around 400 ms (LP) in detected change trials, as compared with undetected change trials. However, the VAN and LP components did not differ as a function of the validity or the cues, suggesting that voluntary spatial perceptive attention to cued items, even though it may facilitate the pre-change display encoding, does not seem to modulate change-detection ERP components.

Conversely, using different paradigms such as masking or rapid serial visual presentation (RSVP), it has been shown that VAN and LP may be differentially modulated by spatial and non-spatial attention. Specifically, it has been observed that the early part of VAN (130–200 ms) seems to be independent of both non-spatial and spatial attention under certain conditions, whereas the later part of VAN (200–300 ms) and the LP (350–700 ms) seem to depend strongly on both spatial and non-spatial attention (see Koivisto and Revonsuo, 2007, 2008a,b).

In summary, it has been observed that exogenous and endogenous spatial attention deployed to the pre-change display using cueing paradigms can improve change detection performance. Although change detection paradigms have not shown dissociations (or attentional effects) in awareness-related ERP components (i.e., VAN, LP), other experimental approaches have shown that while the VAN, proposed as a correlate of phenomenal awareness, seems to be relatively independent of attention, the LP component, proposed as an electrophysiological correlate of access awareness, seems to be largely affected by both spatial and non-spatial perceptual (pre-cued) attention.

As stated before, the Posnerian paradigm has been essential to demonstrate that directing attention to particular locations enhances subsequent encoding and performance for targets appearing at the cued locations. However, our daily activities do not only depend on how we orient our spatial attention to external world, but also depend on how we manipulate the information stored in our mental representations of it. In order to study the orienting of attention to the contents of working memory representations, Griffin and Nobre (2003) designed a paradigm that combined the traditional cueing paradigm (Posner, 1980) with the “partial-report paradigm” (Sperling, 1960). These studies have traditionally employed central informative cues presented after the appearance of a stimulus array (retro-cue) during a delay period, indicating retrospectively the location of the relevant stimuli. Behavioral results derived from these studies have shown that retro-cues significantly optimize accuracy and reaction time in adults in a similar fashion that traditional pre-cues do (see Lepsien and Nobre, 2007; Duarte et al., 2013). It has been suggested that retro-cues might reduce working memory (WM) load. Since items maintained in working memory compete for attentional resources, this type of cueing would specially benefit high load working memory tasks, in which inter-item interference is high (Lepsien and Nobre, 2007). In this sense, retro-cueing would benefit behavior by reducing the number of comparisons needed to perform between targets and their existing memory representations (Makovski et al., 2008). The majority of retro-cueing studies have typically used central endogenous valid retro-cues in target detection tasks. Although previous studies that investigated the exogenous cueing benefit during visual short term memory maintenance showed inconsistent results (Makovski and Jiang, 2007; Sligte et al., 2008; Berryhill et al., 2012; Murray et al., 2013; Pertzov et al., 2013; Shimi et al., 2014), it has been recently shown that exogenous (peripheral) cueing can be also used to control selective attention during memory maintenance (Matsukura et al., 2014).

The electrophysiological studies that have employed this paradigm have mainly explored the ERP correlates of retro-cueing effects during the delay period in working memory tasks. In such studies, a negative deflection with higher amplitude at posterior scalp electrodes contralateral to the visual hemifield where the stimuli are presented has been observed (contralateral delay activity or CDA, see Vogel and MacHizawa, 2004; McCollough et al., 2007; Anderson et al., 2011). Conversely, the ERP studies that have explored the effect of endogenous retrospective attention on the ERP components related to target detection are scarce, and they have only explored late electrophysiological correlates within the P3 latency window after target onset (Duarte et al., 2013). Moreover, the electrophysiological effects of peripheral retro-cueing remain to be investigated.

Behavioral retro-cueing studies have proved that both endogenous and exogenous attention modulates performance, and it has been shown that endogenous retro-cueing modulate late ERP-components elicited by targets. However, exogenous retro-cueing effects on change detection ERP components have not been explored so far. Therefore, in the present study, we used behavioral and electrophysiological measures to study whether exogenous peripheral cues presented during the delay period (retro-cues), that is between the first array and the second array in a one-shot change detection task, modulate the ERP correlates of visual awareness. If visual awareness is independent of peripheral retro-cueing, its ERP correlates should not differ between valid and invalid conditions but only between CD and CB conditions. Otherwise, these ERP correlates would show differences between validly and invalidly cued conditions.

## Materials and methods

### Participants

Twelve young volunteers (3 men, 23–37 years of age, 26.08 ± 3.59) participated in the study. All reported normal or corrected to normal visual acuity as well as no history of neurological or psychiatric condition. The study was performed in accordance with the ethical standards established in the Declaration of Helsinki. Informed consent was obtained from all participants.

### Stimuli and procedure

**Figure 1:**
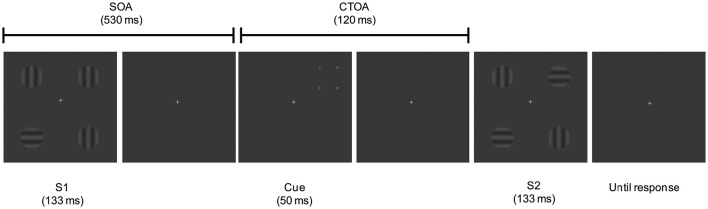
**Schematic illustration of the task**. Trial with a retro-cue signaling the valid location of a change in S2.

Participants were sitting in an armchair in an electrically shielded; sound attenuated, and dimly lit room at a viewing distance of 100 cm from a 21-inch video CRT monitor (1024 × 768 at 70 Hz), with a response pad under their hands. Stimuli consisted of displays containing four sinusoidal gratings (25% contrast, 2.6 cpd, 1° visual angle, 3.5 cd/m2), each one oriented either vertically or horizontally, displayed on a gray background (2.85 cd/m2), and located at a distance of 5° from a central fixation cross, which subtended 0.5 × 0.5° of visual angle. The fixation point remained continuously present until the second display disappeared (see Figure 1). We employed a one-shot change detection paradigm in combination with a spatial peripheral retro-cueing task.

As shown in Figure 1, on each trial, two displays (S1, S2) containing the four sinusoidal gratings were presented successively for 133 ms each and separated by an interval of 650 ms. Each trial started with a 2500 ms fixation presentation. After the appearance of the first display (S1) a delay with a SOA of 530 ms was presented, and followed by a peripheral four-dot cue briefly appearing (50 ms) at one of the four positions where the gratings of S1 appeared previously. After that, the second display (S2) was presented. This display could contain one of the four gratings rotated 90° or could be identical to S1. The time interval comprised between the cue onset and the target onset (S2), or cue-to-target onset asynchrony (CTOA), was of 120 ms.

The experiment consisted of 720 trials, with 30 trials per block. In the 34% of the trials both displays were identical (no-change trials). In the remaining 66% of the trials, any one of the four gratings was replaced by a 90° rotated grating, whereas the other three remained identical across displays (change trials). Exogenous cues predicted 50% of change trials. Therefore, the cues signaled the location of change in a total of 240 trials (valid trials thereafter) and the opposite hemifield in the remaining 240 trials (invalid trials thereafter). Changes on any item occurred in random order and with equal probability. Change and no-change, and valid and invalid trials were randomly intermingled. Percentage of non-change trials was lower than change trials in order to obtain an equal number of trials in each condition (i.e., 240). Similar or even lower non-change percentage of trials has been previously employed in pre-cueing change detection tasks (see Koivisto and Revonsuo, 2005).

Participants were given verbal instructions to maintain their gaze on the central fixation cross and to report whether they had noticed or not a change between the S1 and S2 displays by pressing one of two possible response keys under their left or right hand at the end of each trial. This led to six potential response categories: change detection (CD) and change blindness (CB) both in valid and invalid trials, no-change correct detection (NCC), and false alarms (FA). Speed and accuracy were equally encouraged. Participants were also informed that the cue was irrelevant to perform the task since it did not provide any information about change location. Assignment of response buttons to change or no-change detection was counterbalanced across participants. Subjects were allowed short, self-paced breaks between blocks. Stimuli display and behavioral response collection were carried out using Presentation software (Version 12).

### Electroencephalographic (EEG) recording

Continuous EEG activity was recorded with a Brain Vision Recorder (Brain Products, Inc.) from 60 scalp Ag-AgCl electrodes placed according to the extended 10/20 International System. The cephalic electrodes were referred to the nose tip and grounded with an electrode placed at 10% of the nasion-inion distance above nasion. Vertical and horizontal electrooculogram (EOG) were recorded from above and below the participant's left eye and from the outer canthi of both eyes, respectively. Electrode impedances were kept below 10 kΩ. Sampling rate was 500 Hz/channel. EEG signal was continuously amplified (10 K) and filtered online with a band pass of 0.01–100 Hz.

### EEG data analysis

Vision Analyzer Software (version 2.0, Brain Products, Inc.) was used for off-line processing. The continuous EEG signal was digitally filtered with a band-pass of 0.1–30 Hz. Filtered EEG was segmented into epochs of 2,200 ms to obtain the ERPs for each participant and condition separately. Although epochs were extracted from −200 to 2000 ms relative to S1 presentation in order to obtain a baseline free from artifact activity, analyses focused on electrophysiological activity after S2 onset.

Ocular artifacts associated with blinks and vertical eye movements were removed from the EEG employing the Gratton et al. (1983) method, and EEG epochs exceeding ±100 μV, and/or containing horizontal eye movements were rejected and excluded from averaging. Epochs associated with RTs slower than 1,550 ms were also excluded from averaging, which resulted in a minimum of 85 trials per condition for each subject. Epochs were averaged and baseline corrected (−200 ms) in all active channels.

Since correlates of visual awareness overlap with classical ERP components (i.e., N1–N2, and P3), differences between conditions are most clearly observed in subtraction waveforms. Therefore, ERPs elicited by change blindness in valid conditions were subtracted from the ERPs elicited by detected changes (at both valid and invalid conditions). Subtraction waveforms of ERPs elicited in valid minus invalid change detection conditions were also obtained.

### Statistical analyses

#### Behavioral data analyses

Reaction times (RTs) and change detection accuracy (%) were computed for all participants in all conditions (see Table 1). For behavioral data analyses, CD accuracy was submitted to a Paired-Samples Student's *T*-Test. The accuracy scores in valid and invalid CD conditions were also converted into d' scores (the normalized proportion of correct hits minus the normalized proportion of false alarms) and were submitted to a Paired-Samples Student's *T*-Test. RT were separately entered into repeated measures two-way analyses of variance (ANOVA) with Condition (CD, CB) and Cue (Valid, Invalid) as factors. *Post-hoc* tests were used for correction of multiple comparisons effects by means of Bonferroni procedure when appropriate.

**Table 1:** **Accuracy (%) and RT (ms) across conditions**.

	**Accuracy (%)**		**Reaction time (ms)**	
	**Mean**	**Std dev**	**Mean**	**Std dev**
Change detection valid	62.69	18.47	651.96	115.14
Change blindness valid	37.32	18.47	720.75	126.47
Change detection invalid	37.67	15.96	748.44	102.31
Change blindness invalid	62.33	15.96	688.46	131.16
No change correct	85.27	12.34	670.08	108.56
False alarms	14.73	12.34	753.03	155.71

#### ERP data analyses

To differentiate the electrophysiological activity related to CD from that related to CB during valid and invalid retro-cueing, we performed comparisons between trials containing a change as a function of whether participants reported to be aware of it (CD trials) or reported not to have seen it (CB trials). A blind method was employed to identify the time intervals and the electrodes with significant amplitude differences between conditions, since the use of an a priori hypothesis could have reduced power to detect the effects due to the variations of the present experiment has in relation to previous CB studies. Although data-driven methods have been criticized because they can substantially inflate Type I error rate, here we employed a method that uses a mass univariate approach with *post-hoc* correction for multiple comparisons which allows to control for these type of error (see Luck and Gaspelin, 2017).

ERP waveforms across all electrodes for each participant and condition were submitted to spatiotemporal analyses with BESA Statistics Software (v2.0, July 2015; BESA GmbH, Inc., https://www.besa.de/products/besa-statistics/besa-statistics-overview/). ERP data were first analyzed with repeated-measures univariate ANOVAs with the factor condition (CD, CB in valid, and invalid conditions). *F*-tests were calculated for time points after S2-onset and were subsequently subjected to non-parametric testing. Pair-wise *post-hoc* permutations tests were calculated using Scheffé tests, in order to identify clusters. One thousand permutations were performed with 4 cm distance criteria between neighbor electrodes. Results are considered corrected for multiple comparisons as only those clusters will be identified that have higher values than 95% of all clusters derived by random permutation of data. Additionally, and to ensure that differences were not simply because of the production of a correct response, activity where observers correctly reported that no change had occurred with that related to CD (the NCC condition) was also included in the analyses. Owing to its low rate, the FA condition was discarded from subsequent analyses, as it was not possible to have a number of trials for each participant comparable to that from the other conditions.

In addition to the non-parametric statistical analyses, and in order to test for possible interactions between factors, data were further explored using repeated measures ANOVAs with Condition (CD, CB) and Cue (Valid, Invalid) as within-subject factors. Time and electrode clusters were adjusted at common time-windows and electrodes where cluster *post-hoc* corrected-permutation analyses revealed significant differences between conditions. When necessary, Bonferroni correction for multiple comparisons was used. The level of significance was established in alpha 0.05 for all analyses.

Accordingly, mean amplitudes were averaged across electrode clusters at N2 (228–292 ms), N2-P3 (323–413 ms), and P3 (420–580 ms) latency ranges as follows: N2 [O2/1, Oz, PO7/8, P5/6, P7/8, TP8]; N2-P3 [O1/2, PO7/8, PO3/4, P7/8, P5/6, CP5/6, C5/6, CP3/4, TP7/8, T7/8, P3/4]; P3-parieto-occipital [O1/2, Oz, POz, PO7/8, CP5/6, PO3/4, P7/8, P5/6, P3/4, P1/2, Pz, CP3/4, CP1/2, CPz, TP7/8] and P3-centro-frontal [T7/8, C5/6, C3/4, C1/2, Cz, FC5/6, FC3/4, FC1/2, FCz, FT7/8, F5/6, F3/4, F1/2, Fz]. See **Figure 4**.

## Results

### Behavioral results

**Figure 2:**
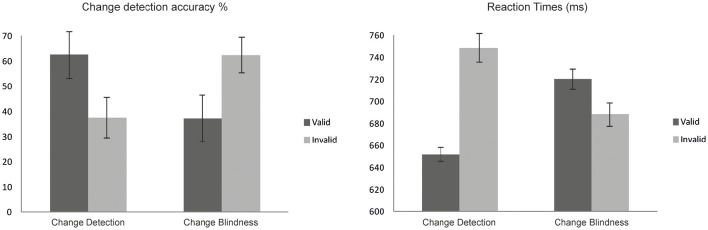
**Change detection (CD) performance**. Valid exogenous retro-cueing decreased RTs and increased accuracy rates in CD.

Behavioral results are depicted in Table 1 and in Figure 2. Overall, the one-shot task designed was successful in achieving an adequate proportion of trials where a change was missed (CB), whereas false alarm percentages (FA) remained relatively low when no change occurred (14.73%).

Paired-Samples *T*-tests revealed that CD was larger in validly cued than in invalidly cued trials [*t*_(11)_ = 7.761, *p* < 0.0001]. In line with these results, sensitivity measure analyses showed that d′ scores were higher [*t*_(11)_ = 8.393, *p* < 0.0001) for validly (1.57 ± 0.51) than for invalidly (0.87 ± 0.55) cued trials.

Analyses on RT values also revealed a significant main effect of Cue [*F*_(1, 11)_ = 32.07, *p* < 0.0001] since valid trials showed reduced RTs compared to invalid trials. Analyses also revealed a Cue by Condition interaction [*F*_(1, 11)_ = 53.65, *p* < 0.0001]. As revealed by *post-hoc* analyses participants were fastest when responding to a correctly detected change in valid trials (*p* < 0.0001) and slower when failing to detect a change in S2 displays (CB) in valid trials (*p* < 0.008). See Figure 2.

### ERP results

**Figure 3:**
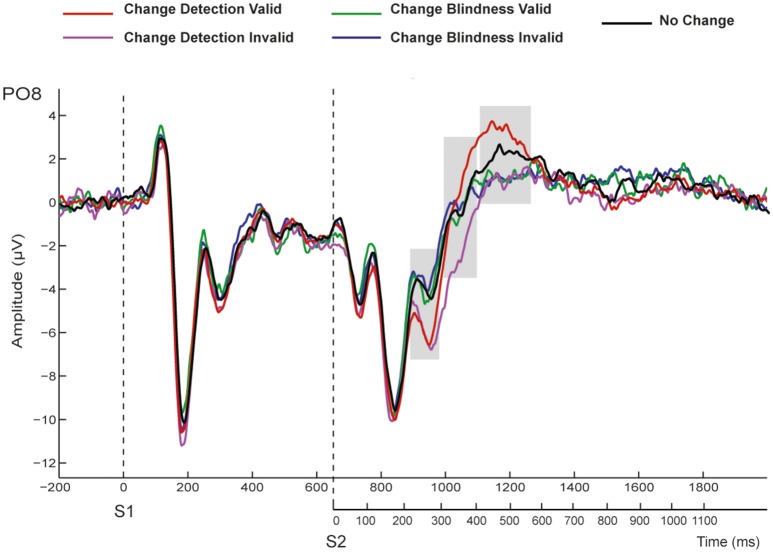
**Grand-averaged ERPs at the representative PO8 electrode for valid and invalid trials in CD, CB, and NCC conditions**. Time windows used for statistical analyses are shaded.

The ERPs elicited by the first display (S1) were dominated by a positive (P1, ~115 ms) and two negative (N1, ~180 ms, and N2 ~300 ms) deflections, both prominent at occipital and parieto-occipital electrodes (Figure 3). Similar components emerged after S2 onset although in this case they were followed by an additional large positivity (P3, ~550 ms) that was maximal at parieto-occipital sites, and clearly prominent in the CD condition for valid trials. As stated before, although epochs were extracted relative to S1 onset, the following reported results correspond to analyses of change-related activity elicited after S2 onset. See, in Figure 3, shaded time-ranges.

#### Non-parametric cluster permutation tests

The results of the cluster-based permutation analyses showed significant differences between CD and CB conditions in several time intervals and electrodes as specified in Table 2. No significant differences were observed when valid and invalid CB conditions were compared. No differences were found between NCC and CB trials.

**Table 2:** **Time intervals post-S2, and the corresponding electrode clusters where permutation analyses (*post-hoc* corrected) showed significant differences between conditions**.

	**Time interval**	**Electrodes within cluster**	
Change detection valid vs. change blindness valid	88–112 ms	C2, C4, C6, CP2, CP4, CP6, CPz, FC4, O2, P2, P4, P6, P8, PO4, PO8, TP8	*p* < 0.001
	228–292 ms	O1, O2, Oz, P5, P6, P7, P8, PO7, PO8, TP8	*p* < 0.002
	450–568 ms	AF3, AF4, AF7, C1, C2, C3, C4, C5, C6, CP1, CP2, CP3, CP4, CP5, CP6, CPz, F1, F2, F3, F4, F5, FC1, FC3, FC4, FC5, FC6, FCz, FP1, Fz, O1, O2, Oz, P1, P2, P3, P4, P5, P6, P7, P8, PO3, PO4, PO7, PO8, POz, Pz, T7, T8, TP7, TP8	*p* < 0.0001
Change detection valid vs. change blindness invalid	422–604 ms	AF3, AF4, C1, C2, C3, C4, C5, C6, CP1, CP2, CP3, CP4, CP5, CP6, CPz, Cz, F1, F2, F3, F4, F5, F7, FC1, FC2, FC3, FC4, FC5, FC6, FCz, Fz, O1, O2, Oz, P1, P2, P3, P4, P5, P6, P7, P8, PO3, PO4, PO7, PO8, POz, Pz, T7, TP7, TP8	*p* < 0.0001
Change detection valid vs. change detection invalid	344–568 ms	AF3, AF4, C1, C2, C3, C4, C5, C6, CP1, CP2, CP3, CP4, CP5, CP6, CPz, Cz, F1, F2, F3, F4, F5, F6, F8, FC1, FC2, FC3, FC4, FC5, FC6, FCz, FP1, FP2, FT7, FT8, Fz, O1, O2, Oz, P1, P2, P3, P4, P5, P6, P7, P8, PO3, PO4, PO7, PO8, POz, Pz, T7, T8, TP7, TP8	*p* < 0.0001
Change detection invalid vs. change blindness valid	340–386 ms	AF4, AF8, C3, C6, CP5, CP6, F1, F2, F3, F4, F6, F8, FC1, FC3, FC4, FC5, FC6, FP2, FT8, Fz, O1, O2, Oz, P5, P6, P7, P8, PO3, PO4, PO7, PO8, T7, T8, TP7, TP8	*p* < 0.001
Change detection invalid vs. change blindness invalid	304–440 ms	AF3, C1, C2, C3, C4, C5, C6, CP1, CP2, CP3, CP4, CP5, CP6, CPz, F1, F2, F3, F4, F6, F7, F8, FC1, FC2, FC3, FC4, FC5, FC6, FT7, FT8, O1, O2, Oz, P1, P2, P3, P4, P5, P6, P7, P8, PO3, PO4, PO7, PO8, POz, Pz, T7, T8, TP7, TP8	*p* < 0.0001
Change detection valid vs. no change correct	256–270 ms	P8, PO8, TP8	*p* < 0.009
	486–526 ms	CP3, CP5, P1, P3, P5, PO3, Pz	*p* < 0.009
Change detection invalid vs. no change correct	336–478 ms	C1, C3, C5, CP1, CP3, CP5, F7, FC1, FC3, FC5, FT7, O1, O2, Oz, P1, P3, P4, P5, P6, P7, P8, PO3, PO4, PO7, PO8, POz, Pz, T7, TP7, TP8	*p* < 0.0001
	402–480 ms	C6, CP4, CP6, F8, FC6, FT8, P6, P8, PO8, T8, TP8	*p* < 0.002

#### Parametric tests

##### N2 latency range

**Figure 4:**
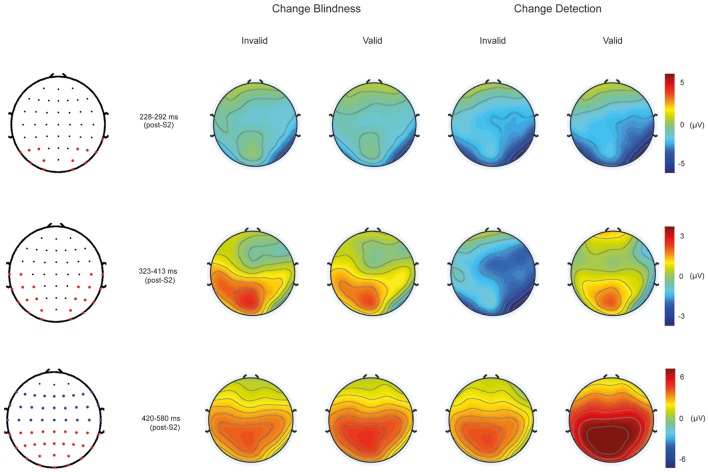
**Scalp maps showing the topographical distribution from 228 to 292 ms post-S2 (Top)**, from 323 to 413 ms post-S2 **(Middle)**, and from 420 to 580 ms post-S2 **(Bottom)** in CD and CB conditions. Map with red dots at lateralized parieto-occipital electrodes indicates the electrode clusters employed to parametrically test statistical differences among conditions within the N2 and N2-P3 latency ranges. Map with fronto-central blue and parieto-occipital red dots indicates the electrode clusters used to test statistical differences in the P3 latency range.

ANOVA at the N2 latency range at posterior sites (228–292 ms) revealed significant effects of Condition [*F*_(1, 11)_ = 9.56, *p* < 0.01]. As can be appreciated in Figure 4, CD conditions showed larger negative amplitudes (mean: −3.9 ± 1.8 μV) than CB conditions (mean: −2.5 ± 0.986 μV; see Figure 4, upper panel).

##### N2-P3 latency range

ANOVA using the same within-subject factors at the 323–413 ms showed significant main effects of Cue [*F*_(1, 11)_ = 12.19, *p* < 0.005; mean valid: −0.352 ± 0.57 μV; mean invalid: −0.451 ± 0.545 μV]. Significant interactions were found between Condition and Cue [*F*_(1, 11)_ = 8.164, *p* < 0.016]. Bonferroni corrected *post-hoc* tests showed significant differences between CD (−1.77 ± 0.72 μV) and CB (0.86 ± 0.76 μV) conditions in invalid trials (*p* < 0.025), and between valid (−0.13 ± 0.73 μV) and invalid (−1.77 ± 0.72 μV) CD conditions (*p* < 0.008; See Figure 4, middle panel).

##### P3 latency range

The ANOVA performed at the P3 latency range (420–580 ms) at posterior regions (see Figure 4, bottom-left map, red dots) revealed a significant main effect of the Cue factor [*F*_(1, 11)_ = 20.16, *p* < 0.001] that was due to a larger amplitude in valid (4.06 ± 0.74 μV) than in invalid trials (2.46 ± 0.64 μV). A Condition by Cue interaction was also significant [*F*_(1, 11)_ = 12.22, *p* < 0.005]. *Post-hoc* Bonferroni corrected tests revealed that this effect was driven by the significantly greater mean amplitude values to CD in valid trials (5.19 ± 0.83 μV) than in invalid trials (2.43 ± 0.57 μV, *p* < 0.0001); this interaction was also due to the greater amplitude in CD valid trials (5.19 ± 0.83 μV) compared to CB valid trials (2.94 ± 0.7 μV, *p* < 0001).

At centro-frontal regions, ANOVA at the P3 latency range (420–580 ms; see Figure 4, bottom-left map, blue dots) also revealed a main effect of Cue [*F*_(1, 11)_ = 8.35, *p* < 0.015] that was again due to larger main amplitudes in valid (3.04 ± 0.48 μV) than in invalid trials (1.99 ± 0.41 μV). A significant interaction effect was also found between Condition and Cue [*F*_(1, 11)_ = 14.23, *p* < 0.003]. *Post-hoc* Bonferroni corrected analyses revealed again larger amplitudes in valid (3.75 ± 0.55 μV) than in invalid CD trials (1.91 ± 0.37 μV, *p* < 0.001); and in valid CD trials (3.75 ± 0.55 μV) compared to valid CB trials (2.32 ± 0.46 μV, *p* < 0.001). See Figure 4, bottom-left map, blue dots.

**Figure 5:**
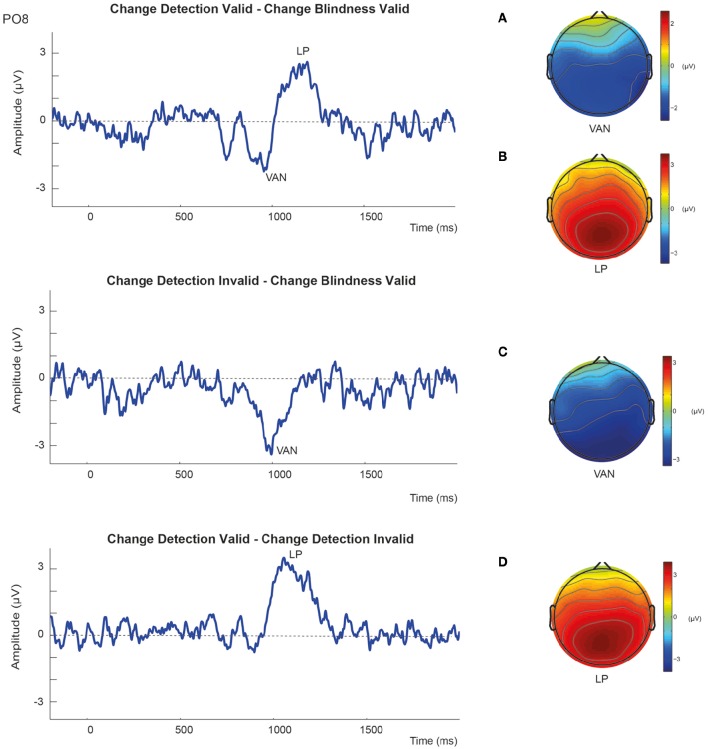
**Subtraction waveforms at the representative PO8 electrode together with corresponding scalp maps showing the topographic distribution of (A,C)** the Visual Awareness Negativity (VAN); and **(B,D)** the Late Positivity (LP).

In summary, the results showed that detected changes elicited more negative amplitudes than undetected changes in the N2 latency range independently of the validity of the retro-cue. Alternatively, amplitudes in the P3 time window were more positive to detected changes than to undetected changes, although this effect seemed to be associated with correct change detection in validly cued trials. This can be more easily seen in the difference waveforms. As it can be observed in Figure 5, when ERPs to valid CB trials were subtracted from those to valid CD trials, awareness of a change was associated with a negative amplitude enhancement around N2 latency window after S2 onset (VAN component) that was followed by an enhanced positivity in the P3 time window (LP). However, the reported differential negative component (VAN) was not followed by the positive enhancement (LP) when valid CB ERPs were subtracted from invalid CD. Moreover, when invalid CD trials were subtracted from valid CD trials, the resulting waveforms did not show any negativity suggesting the existence of a VAN, and only the LP component could be observed.

## Discussion

The objective of the present research was to study the effects of attention on the behavioral and electrophysiological correlates of visual awareness (i.e., VAN, LP) in a change detection task by using exogenous cues presented during the delay period.

### Behavioral results

The results of the present study, at a behavioral level, showed that subjects were significantly faster (shorter reaction times) and more accurate (higher percentage of correct change detection) in trials with valid retro-cues than in trials with invalid retro-cues. Therefore, these findings showed significant behavioral effects of retro-cueing even when using peripheral exogenous cues in a change detection task (Figure 2).

Similar behavioral effects have been reported in working memory capacity in the retro-cueing literature across a large diversity of stimuli, timing, set sizes, and experimental designs (i.e., Griffin and Nobre, 2003; Landman et al., 2003; Lepsien et al., 2005, 2011; Lepsien and Nobre, 2006, 2007; Nobre et al., 2006, 2008; Makovski and Jiang, 2007; Matsukura et al., 2007; Makovski et al., 2008; Sligte et al., 2008, 2009, 2010, 2011; Berryhill et al., 2012; see Tanoue and Berryhill, 2012). Moreover, studies that have compared pre- and retro-cueing benefits have shown similar improvements associated with orienting perceptual (i.e., pre-cueing, ~14–19% facilitation) and internal (i.e., retro-cueing, ~11–17% facilitation) attention (Griffin and Nobre, 2003; Nobre et al., 2004). See Souza and Oberauer (2016) for a recent review.

Most of the research performed in this context has typically used central endogenous valid retro-cues (i.e., arrows, line segments). However, less attention has been paid to the influence of cue type (central vs. peripheral) and validity (i.e., cue reliability) on the magnitude of the retro-cueing effects. Berryhill et al. (2012) tested the behavioral effects of four retro-cue conditions: neutral cue, arrow retro-cue, dash retro-cue, and number retro-cue. The arrow, dash, and number cues were always 100% informative cues. Results showed that the retro-cue effect was strongest on trials presenting the arrow retro-cue. Consistent with Berryhill et al. (2012) findings, Shimi et al. (2014) showed that highly reliable cues (i.e., 100% valid) increased performance in target detection tasks. However, the reduction of validity of cues down to 50% only left a residual advantage in the accuracy and response times for target probes at validly cued locations compared to neutral trials. In a study performed by Pertzov et al. (2013) participants were briefly presented with randomly oriented colored bars and, after a variable delay, were asked to reproduce from memory the orientation of one of the bars, specified either by its location or by its color. Peripheral and central retro-cues correctly predicted the probed memory item with 70% validity. Their results showed that, both central and peripheral retro-cues similarly protected selected items in memory from gradual degradation when they were valid. However, the retro-cues made the non-selected items more fragile than selected items in trials without any cue.

Although these behavioral studies showed inconsistent results, a recent work conducted by Matsukura et al. (2014) demonstrated that exogenous peripheral cueing with low validity can actually guide attention to a particular item's location represented in working memory as efficiently as the endogenous (central) cueing. In this study the observers recognized validly cued items more accurately than neutrally cued items by utilizing the cue that appeared at the exact same perceptual location with the cued item, despite the fact that they were non-informative (50% validity). Although peripheral non-informative cues are generally employed to engage exogenous attention, subjects were instructed to take advantage of the cueing information. Due to this manipulation the authors concluded that, unlike the exogenous cueing benefit observed during sensory processing, the observed behavioral benefit was probably generated through a goal-directed selection of attention.

The behavioral results in the present study are in line with those reported by Matsukura et al. (2014). However, there are several differences between both experimental designs: in the present study we employed a change detection task, subjects were explicitly asked to ignore the peripheral cues, and we did not include a neutral condition. Accordingly, future studies should be performed using change detection tasks, and comparing execution results with low-validity, exogenous peripheral cues, with those with neutral retro-cue conditions.

### ERP results

Electrophysiological results of the present study showed that the ERPs elicited after the second display (S2) significantly differed between change detection (CD) and change blindness (CB) conditions around the N2 latency range (228–292 ms) at occipital and parieto-occipital electrodes. Neither main effects of cueing, or condition by cueing interactions were significant in this latency range. This effect was followed by an additional broadly distributed positive difference at the P3 latency range. At both centro-frontal and posterior regions at the P3 latency range (420–580 ms) amplitudes were larger for CD conditions when compared with CB conditions, but only in validly cued trials (Figure 3). These differences were not simply because of the production of a correct response, since correct trials with no change (NCC) differed from valid CD trials at posterior sites in the N2 latency range, and at central, parietal and occipital sites in the P3 latency range (Table 2).

These findings are in agreement with previous studies that have shown that correct change detection elicits a negative deflection in the N2 latency range (i.e., VAN), a component that has been associated with the activation of occipito-temporal regions, and which has been considered a correlate of phenomenal awareness. Although VAN typically occurs at early latencies, in the present study this component showed a later latency, possibly due to differences in the experimental design and stimuli used here (i.e., low contrast stimuli; see Wilemius and Revonsuo, 2007). Validly cued correctly detected changes elicited also larger deflections at the P3 latency range, a late positive component (i.e., LP) which has been shown to reflect the activation of large fronto-parietal and posterior networks that have been associated with access awareness (see Koivisto and Revonsuo, 2008b, 2010; Railo et al., 2011 for reviews).

Subtraction waveforms comparing aware (CD) and unaware trials (CB) revealed more clearly these effects (Figure 5). As it can be observed, successful change detection was associated with the emergence of the VAN independently of the validity of the trial, that is, independent of spatial retro-cueing. Conversely, the LP component was only evident when valid, but not invalid, correct changes were subtracted from change blindness trials, and therefore largely dependent on attentional conditions. Hence, these electrophysiological results showed that (1) VAN and LP components can be dissociated using exogenous retro-cues, and (2) LP, as seen before, does not necessarily constitute a correlate of awareness *per-se* in change detection tasks.

As discussed below, dissociations between VAN and LP components have been also previously shown by other studies having explored the relationship between visual consciousness and selective perceptive attention using different paradigms other than change detection tasks.

#### Non-spatial attentional manipulations

For example, using a backward masking paradigm in combination with a Navon-like task, Koivisto et al. (2006) showed that in the N1-N2 time range at occipital sites (VAN) a larger negative shift was elicited by non-masked stimuli (aware) when compared with masked stimuli (unaware) in local and global attentional conditions. However, the manipulation of non-spatial attention had a stronger influence in the P3 time-range around 400 ms, peaking at parietal sites.

Similarly, it has been shown that the detection of attended target stimuli as well as ignored non-target stimuli elicit VAN, whereas LP is eliminated or greatly attenuated in response to non-targets. In a series of studies, Koivisto et al. (2005) firstly manipulated visual awareness and selective non-spatial attention parametrically in a masking task using stimuli presented in the center of the visual field. Their results showed that awareness and attention interacted in the 200–260 ms time window (late part of the VAN) over the left hemisphere and the temporal lobes, but not in the early part of this negative component (130–200 ms). As stated by the authors, although the awareness-related negativity (VAN) was modified by attention in the 200–260 ms time window (targets elicited larger VAN than non-targets), a strong VAN was also found for non-targets, suggesting that this component is elicited independently of selective attention. On the contrary, they found that mean amplitudes on the P3 latency range (290–700 ms) showed that targets elicited more positive ERPs than non-targets, and ERPs to aware stimuli were more positive than those to unaware ones. Similar results were found by Koivisto and Revonsuo (2008b) using a similar masking task along with a passive task, and also showed that the VAN (100–300 ms) was present (although reduced) when the participants were not required to attend to the stimuli. Accordingly, the authors concluded that the neural processes responsible for visual awareness (VAN) are initially independent of selective feature-based attention, but they seem to be modulated by attention after 200 ms, whereas LP shows a strong dependence on selective attention to features.

#### Spatial and non-spatial attentional manipulations

In a later study, Koivisto and Revonsuo (2007) manipulated both non-spatial and spatial attention again employing a masking task. Analyses of difference waveforms of unmasked/aware vs. masked/unaware conditions showed that in the 120–290 ms time range (VAN) non-differences were observed between targets and non-targets or between attended and unattended visual fields. However, analyses in the P3 latency range (290–700 ms) showed that attended targets and stimuli presented in the attended hemifields elicited larger amplitudes. Since this study employed unilateral stimulation, the authors conducted a similar study employing bilateral stimulation. In their study, Koivisto et al. (2009) tested whether phenomenal consciousness of a stimulus could not occur without any spatial attention to the region in the stimulus field where the unattended stimulus appears. The electrophysiological results from the attended visual field replicated their earlier findings regarding non-spatial selection of features and objects. However, in the unattended field no reliable early electrophysiological correlates for visual awareness (VAN) were observed, suggesting that attention must be focused on the stimuli for VAN to emerge. Again, the later stages of conscious processing, as indicated by the LP between unmasked and masked conditions in the P3 time window (350–700 ms) depended strongly on both spatial and non-spatial selection.

In summary, these studies have shown that although the early part of VAN (130–200 ms) seems to be independent of non-spatial attention, spatial attention seems to be a prerequisite for this component to be elicited. On the other hand, the later part of VAN (200–300 ms) and the LP (350–700 ms) seem to depend on both spatial and non-spatial perceptive attention. Moreover, they have proved that it is possible to observe VAN without LP in conditions where the stimuli are consciously perceived, suggesting that LP is not a necessary correlate of visual awareness.

In our study, we found that at posterior locations within the latency range between 228 and 292 ms, VAN was independent of peripheral retro-cueing. Therefore, even if perceptive spatial attention is a prerequisite for this difference to occur, spatial retro-cueing does not modulate VAN, even at latencies where both spatial and non-spatial attentional effects have been found to affect this component using backward masking tasks. Nevertheless, it should be mentioned that masking paradigms such as those used by Koivisto et al. (2005, 2006, 2009) and Koivisto and Revonsuo (2007, 2008b), differ in many aspects from change detection tasks; from those aspects it is remarkable the fact that masking tasks may produce an enhancement of negativity to targets that represents an electrophysiological response reflecting non-spatial selection. Moreover, there are some contradictory findings with regard to the author's conclusion on the effects of spatial attention on VAN since they found that VAN did not differ between the attended and unattended visual fields in Koivisto and Revonsuo (2007) study.

More consistent with previous findings are the modulatory effects found here in the LP component. Although many studies have observed only the late component when comparing consciously detected stimuli with undetected stimuli (Niedeggen et al., 2001; Fernandez-Duque et al., 2003; Lamy et al., 2009; e.g., Chica et al., 2010), LP does not directly correlate with explicit detection, but it might be related to the P3 component, and reflect postperceptual operations such as evaluation of the stimulus as relevant for task purposes (e.g., Donchin and Coles, 1988; see Railo et al., 2011). In this line, it has been proposed that the LP may index differences in confidence ratings rather than perceptual processes involved in the processing of the change, since P3 amplitudes have been found largest for correctly identified changes, and smaller P3 effects have been found for merely detected changes and also for false alarms (Koivisto and Revonsuo, 2003; Eimer and Mazza, 2005; although see Salti et al., 2012 for a different interpretation). Moreover, Busch et al. (2010) studied ERP effects of visual change processing (as compared to change blindness) when observers merely detected the presence of a change (“sensing”) and when they identified the changing object in addition to detection (“seeing”). Although the VAN was similar for their “sensing” and “seeing” conditions, no LP was recorded in the “sensing” condition but only in the “seeing” one.

Results found in the present study are difficult to discuss within the context of retro-cueing research, since only one ERP study has evaluated the effects of retrospective attention on target related components but employing a working memory task. Duarte et al. (2013) performed such study employing central informative (100%) retro-cues that were presented during the maintenance period (between the study and the probe stimuli). Their results showed that the P3b elicited to correctly detected targets peaked later in no cue trials (~60–90 ms) and was more short-lived in young adults. Studies within the context of working memory have generally interpreted the P3b component elicited by target stimuli following the widely recognized “context updating” theory (Donchin and Coles, 1988; Polich, 2007). Specifically, the “context updating theory” states that after the target is processed at a sensory-perceptual level, it is evaluated by comparing its representation in working memory with that of the other stimuli included in the task. If this comparison process results in a matching between the actual target and its representation, a P3b component is elicited, and the contents of working memory are updated in order to maintain the correct execution of the task. Thus, when a memory representation has been more recently and successfully processed, and it is active in working memory, less updating is needed and the latency of P3b is reduced while its amplitude is increased (see Kok, 2001; Polich, 2007 for reviews). Following the central prediction of this theory, Duarte et al. (2013) interpreted their findings as suggesting that retrospective attentional cueing may enhance working memory performance by reducing the degree of probe-related evaluation and updating during test.

Therefore, and as reported in previous studies, the modulations by retrospective attention on LP amplitude shown here may suggest that this late component might be probably associated with some of the above mentioned P3 related process, and they may reflect a gain process when attention is validly guided to the correct representation of the detected change in working memory.

There are limitations in the present study. First, an alternative interpretation may explain the modulations observed in the VAN and LP components. As suggested by one of the reviewers, cued trials could launch a bottom-up stimulus-driven attentional modulation of the comparison process instead of an exogenous modulation of attention within working memory. This could be proved by studying the modulation of the early components of the S2-related ERP and by analyzing working memory-related components such as CDA. However, our change detection paradigm does not allow the study of the ERP related activity between the retro-cue onset and target-related ERP responses since the CTOA employed was too short. Accordingly, further studies should be conducted in order to study the time course of exogenous retro-cued attention in change blindness ERP studies by enlarging CTOAs, which could allow the study of working memory sensitive components such as the CDA (see for example Williams and Woodman, 2012).

Interestingly, our results resembled the findings observed by Hopfinger and Mangun (1998, 2001) using the classical exogenous attentional perceptive paradigms (i.e., with peripheral pre-cues). As observed here, they reported that the P3b amplitude was larger for cued-location targets (valid) relative to uncued-location targets (invalid), at short CTOA. The authors suggested that reflexive attention mechanisms caused cued location stimuli to be treated as more salient or potentially significant at higher stages of stimulus evaluation, even when both cued and uncued locations are known by the participant to be equally frequent and equally relevant. Therefore, the amplitude modulations of the LP component observed here might be related to reflexive internal orienting mechanisms, which caused cued-location stimuli to be treated as more relevant at higher states of stimulus evaluation during change detection and its locations processed in a more relevant fashion.

Although there is currently some discussion regarding the degree of similarity between attentional shifts of perceptual and internal attention, and it has been suggested that internal attention may be less flexible than perceptual attention (Tanoue and Berryhill, 2012), separable although partially overlapping mechanisms may be shared by both types of attention (Lepsien and Nobre, 2006 for reviews; see Gazzaley and Nobre, 2012). Further studies should be conducted in other to characterize possible differences among perceptual and internal involuntary attention. In conclusion, present results confirm previous behavioral findings that show that exogenous retro-cues during the delay interval facilitate subsequent change detection. This effect seems to be present even when subjects are asked to ignore the cues. The present electrophysiological results showed that VAN and LP components could be dissociated through manipulations of exogenous spatial retro-cueing using a change detection task. VAN component was observed when comparing change blindness condition (unaware) with correctly detected changes (aware) in all attentional conditions (i.e., validly and invalidly retro-cued trials). These results suggest that, unlike the modulatory effects observed during perceptive spatial selection, VAN seems independent of exogenous retro-cueing and therefore phenomenal awareness does not seem to be modulated by this type of attention. Alternatively, LP was not observed when unaware conditions were compared with those where, although subjects were aware of the changes, the retro-cues invalidly signaled the location of the change. This effect suggests, as seen in previous studies with other than change detection tasks, that LP might not be associated to awareness *per-se* and, as it has been observed using perceptive attentional manipulations, exogenous retro-cueing also modulate the access or reflective awareness proposed to be indexed by this component.

## Ethics statement

The study was conducted according the ethic rules of the Spanish Ministry of Economy and Competitiveness (MINECO) grants, which did not require the ethical approval from a committee since present study (a) did not imply clinical human research, (b) pre-embryo related research, (c) use of tissues or biological samples of human origin, or (d) use of personal genetic information. Moreover, (1) participants of the present study were young volunteers (23–37 years of age) with no history of neurological or psychiatric condition, (2) we did not use invasive methods (i.e., ERP) and therefore the participation did not involve any risk to the subjects, (3) each participant signed the informed consent form before participation, and (4) the study was performed in accordance with the ethical standards established in the Declaration of Helsinki.

## Author contributions

PP designed the experiment, analyzed and interpreted the data, and wrote the manuscript. AR designed the experiment, collected the data and interpreted the results. FG collected and analyzed the data. EA designed the experiment, interpreted data, and critically revised the work.

## Funding

This work was supported by the Spanish Ministry of Economy and Competitiveness (MINECO) grant PSI2014-53743-P.

### Conflict of interest statement

The authors declare that the research was conducted in the absence of any commercial or financial relationships that could be construed as a potential conflict of interest.

## Abbreviations

CB: Change blindness
CD: Conscious detection of the change
CTOA: Cue-to-target onset asynchrony
EEG: Electroencephalography
EOG: Electrooculogram
ERP: Event related potential
FA: False alarms
LP: Late Positivity
NCC: No-change correct detection
VAN: Visual Awareness Negativity.
